# Mapping the distribution of *Loa loa *in Cameroon in support of the African Programme for Onchocerciasis Control

**DOI:** 10.1186/1475-2883-3-7

**Published:** 2004-08-06

**Authors:** Madeleine C Thomson, Valérie Obsomer, Joseph Kamgno, Jacques Gardon, Samuel Wanji, Innocent Takougang, Peter Enyong, Jan H Remme, David H Molyneux, Michel Boussinesq

**Affiliations:** 1 Liverpool School of Tropical Medicine, Liverpool, UK, L3 5QA; 2 Laboratoire mixte Institut de Recherche pour le Développement (IRD) – Centre Pasteur du Cameroun d'Epidémiologie et de Santé publique, Centre Pasteur du Cameroun, BP 1274 Yaoundé, Cameroon; 3 Department of Life Sciences, Faculty of Science, University of Buea, P.O. Box 63, Buea, Cameroon; 4 Department of Public Health, Faculty of Medicine and Biomedical Sciences, University of Yaoundé I, P.O. Box 1364, Yaoundé, Cameroon; 5 Tropical Medicine Research Station, P.O. Box 55, Kumba, Cameroon; 6 UNDP/World Bank/WHO Special Programme for Research and Training in Tropical Diseases (TDR), WHO, Geneva, Switzerland

## Abstract

**Background:**

*Loa loa *has recently emerged as a filarial worm of significant public health importance as a consequence of its impact on the African Programme for Onchocerciasis Control (APOC). Severe, sometimes fatal, encephalopathic reactions to ivermectin (the drug of choice for onchocerciasis control) have occurred in some individuals with high *Loa loa *microfilarial counts. Since high density of *Loa loa *microfilariae is known to be associated with high prevalence rates, a distribution map of the latter may determine areas where severe reactions might occur. The aim of the study was to identify variables which were significantly associated with the presence of a *Loa *microfilaraemia in the subjects examined, and to develop a spatial model predicting the prevalence of the *Loa *microfilaraemia.

**Methods:**

Epidemiological data were collected from 14,225 individuals living in 94 villages in Cameroon, and analysed in conjunction with environmental data. A series of logistic regression models (multivariate analysis) was developed to describe variation in the prevalence of *Loa loa *microfilaraemia using individual level co-variates (age, sex, μl of blood taken for examination) and village level environmental co-variates (including altitude and satellite-derived vegetation indices).

**Results:**

A spatial model of *Loa loa *prevalence was created within a geographical information system. The model was then validated using an independent data set on *Loa loa *distribution. When considering both data sets as a whole, and a prevalence threshold of 20%, the sensitivity and the specificity of the model were 81.7 and 69.4%, respectively.

**Conclusions:**

The model developed has proven very useful in defining the areas at risk of post-ivermectin *Loa*-related severe adverse events. It is now routinely used by APOC when projects of community-directed treatment with ivermectin are examined.

## Background

Knowledge of the spatial distribution of *Loa loa *is important in countries involved in the African Programme for Onchocerciasis Control (APOC) and is also now a significant issue for the Global Programme to Eliminate Lymphatic Filariasis (GPELF). Both programmes rely on the wide-scale distribution of anti-helminthic drugs to poor communities using community-directed drug distribution schemes. A problem was first observed in Cameroon where a series of reports of severe and sometimes fatal encephalopathic reactions to ivermectin (Mectizan^®^) in individuals with high *Loa loa *microfilarial counts was made [1-3]. Similar problems may occur when albendazole (used to control lymphatic filariasis) is distributed in *Loa loa *endemic areas although evidence for this is contradictory [4-7].

*Loa loa *is associated with tropical "eye worm" (migration of adult worms across the sub-conjunctiva), Calabar swelling, oedemas and prurities but is not considered as pathogenic as other filarial worms, and is consequently less well studied. It is transmitted by species of horse-flies (*Chrysops *spp.), most commonly *Chrysops dimidiata *and *C. silacea *which inhabit the forest areas of West and Central Africa, extending to the Ethiopian border. Severe and fatal reactions to ivermectin have been associated with individuals with high *Loa loa *microfilarial loads (more than 30,000 microfilariae (mfs) per ml of blood) and those with more than 50,000 mfs/ml are considered at very high risk [1,8-10].

Changes in the protocols for drug administration and post-treatment surveillance in areas considered to be at-risk of severe reactions to ivermectin have been implemented [11,12], but the detailed geographic distribution of *Loa loa *remains unclear [13]. Such information is essential in terms of developing safe treatment, and above all surveillance strategies across the region and, given the vast area which may be affected, it is recognized that rapid assessment methods must be developed to evaluate the risk of severe reactions in communities co-endemic for loiasis and onchocerciasis.

Recent studies have shown that there is a close relationship between intensity of microfilariae infection and prevalence rates of *Loa loa *[14,15] suggesting that a distribution map based on prevalence of infection alone (and not intensity, which would require time-consuming counting of mfs) would provide sufficient information to delineate areas of high risk of severe reactions. The aim of our study was to develop a map indicating the areas where *Loa loa *infection may be high enough (i.e. with a prevalence of *Loa *microfilaraemia in adults exceeding 20%) that poses an operational problem for drug distribution by the Community-Directed Treatment with Ivermectin (CDTI) strategy [16].

As a preliminary step we created a risk model for *Loa loa *in West and Central Africa based on the relationship of crude *Loa loa *prevalence data (obtained from a literature search) to a wide range of environmental variables. Initial results suggested that land/forest cover derived from NOAA-AVHRR satellite data (i.e. collected by the Advanced Very High Resolution Radiometer on board the satellite series operated by the National Ocean and Atmospheric Administration of the USA), and soil type (from the FAO digitised soil map of Africa), are significant predictors and a preliminary risk map was produced [17]. However, when this first model was tested against field data from Cameroon it was found to poorly represent areas of high risk of infection in certain districts [18]. Possible reasons for this include: (a) the low spatial resolution of the satellite and environmental data used (1 km) which was unable to identify narrow gallery forests in savannah areas and/or (b) the use of prevalence data from various sources which had not been standardised by age or sex.

In order to improve the quality of epidemiological data being used to develop the model, we created a new prevalence database from a series of surveys conducted by the team of the *Institut de Recherche pour le Développement *(IRD) at the *Centre Pasteur du Cameroun *(CPC). A second independent data set, collected as part of a project supported by the UNDP/World Bank/WHO Special Programme for Research and Training in Tropical Diseases (TDR) to calibrate a rapid clinical diagnosis of *Loa loa *(RAPLOA) [15], was used to validate the results of the model.

## Methods

### Epidemiological data

Epidemiological data used to create the model was obtained from a series of field studies undertaken by the IRD-CPC laboratory in Cameroon during the period 1991–2001 in which the presence of *Loa loa *parasites in the blood of individuals was assessed. The data come from five regions: (a) the forest-savannah mosaic area of the Mbam and Kim division (Central province); (b) the degraded forest area of the Lekie division (Central province); (c) the dense forest area of the southern part of the Central province; (d) the highland savannah area of the Western Province; and (e) the savannah areas of the districts of Banyo and Bankim, in the Adamaoua province. The Ministry of Public Health of Cameroon provided ethical approval for these surveys.

The total number of subjects included in the analysis was 14,225 individuals for 94 villages. Study participants consisted of individuals over the age of 5 years who gave their consent or for whom consent was obtained from the parent or guardian. Individuals who had had filaricidal drugs, such as ivermectin or diethylcarbamazine, in the previous five years were not included in the study. The arrival of the research team in the villages was announced to the population one week before the event through the local authorities and teachers. The objective of the examinations were clearly explained, and it was stated that each individuals results would be returned. On the given day, the team settled at a central place of the village (usually at the chief's home, or in a school), and all the volunteers were examined between 10.00 and 16.00 hours. A standardized quantity of capillary blood was obtained with a non-heparinized micro-capillary tube. Prior to 1994, the amount taken was 30 μl, and thereafter, 50 μl. After Giemsa staining, the slides were examined under a microscope and the presence of *Loa *mfs was recorded.

The latitude and longitude of all study villages were obtained from either the ordinance survey map or a global positioning system. Thus the data set included village name, longitude and latitude, alongside data on individuals examined (age, sex, standard size of blood sample taken and presence/absence of *Loa loa *infection). A summary of the epidemiological data used is presented in Table 1.

**Table 1:** Epidemiological data sets used in development of the spatial model for predicting the prevalence of *Loa *microfilaraemia

**Region**	Sex*	**Blood sample (μl)**	**Prevalence of ***Loa loa*	**Age range**	**No. subjects**	**No. villages**
Banyo-Bankim	M	50.00	22.21	5–98	1783	16
Banyo-Bankim	F	50.00	20.00	5–90	1815	
South of Central Province	M	30.00	32.81	5–80	381	5
South of Central Province	F	30.00	27.37	5–80	453	
South of Central Province	M	50.00	24.76	5–86	941	17
South of Central Province	F	50.00	18.49	5–90	1206	
Lekie Department	M	30.00	26.33	5–91	1052	14
Lekie Department	F	30.00	19.10	5–92	1340	
Lekie Department	M	50.00	23.96	5–80	359	3
Lekie Department	F	50.00	21.41	5–90	369	
Mbam et Kim	M	30.00	12.37	5–99	1293	24
Mbam et Kim	F	30.00	4.53	5–95	1281	
Western Province	M	50.00	6.22	15–99	916	15
Western Province	F	50.00	3.76	15–99	1036	

* M = male and F = female

### General principles of the analysis

The aim of the study was to identify variables which were significantly associated with the presence of a *Loa *microfilaraemia in the subjects examined. Besides data on individuals (age, sex, size of blood sample), we investigated whether some variables describing the environment of their place of residence (see list of variables below) were significantly related to the microfilaraemia. For these environmental variables, distance operatives indicating whether or not villages were within 5 km of potential at-risk variables were created. Distance operatives were based on the normal dispersal range of *Chrysops *which has been shown to be within 5 km of their breeding sites [19].

In order to design a valid modelling structure, univariate analysis was first undertaken of the relationship between, on the one side, individual and environmental variables, and on the other side the variable of interest, i.e. the presence/absence of *Loa loa *mfs in the individual. Then we developed a series of logistic regression models (multivariate analysis) to describe variation in the prevalence of *Loa loa *microfilaraemia using individual level co-variates (age, sex, μl of blood taken for examination) and village level environmental co-variates. Logistic regression is useful to predict the presence or absence of a characteristic or outcome based on values of a set of predictor variables. It is similar to a linear regression model but is suited to models where the dependent variable is dichotomous. Logistic regression coefficients can be used to estimate odds ratios for each of the independent variables in the model. In our example the dichotomous variable is the presence or absence of *Loa loa *infection in each individual in the sample. Those variables which did not have a significant relationship with age and sex adjusted prevalence were excluded from further analysis. All variables were analysed at individual level.

### Environmental variables and data sets included in the analysis

The distribution of the human population in sub-Saharan Africa is relatively well described from population census data obtained at the national and sub-national level. The population data for 1990 used in this analysis was obtained from the Global Resource Information Database of the United Nations Environment Programme (UNEP-GRID), Eros Data Centre [20]. These population density surfaces (with 1 km resolution) have been derived from a model including population estimation for countries, population of major urban centres and transportation network and accessibility. They have been used in numerous studies involving population distribution in Africa, including studies on estimating the burden of malaria [21].

The USGS (United States Geological Survey) hydrologic digital dataset provides detailed descriptions of the topography of the area, including elevation (Digital Elevation Model, DEM), slope, aspect (direction of maximum rate of change in elevation between each cell and its eight neighbours and representing direction of slope), flow accumulation (defining amount of upstream area draining into each cell) [22].

Satellite data indicating the 'greenness' of the environment (Normalised Difference Vegetation Index, NDVI) was obtained from the VEGETATION sensor launched in 1998 onboard the SPOT 4 satellite system. NDVI images have been specifically tailored to monitor global land surfaces' parameters on a daily basis with a medium spatial resolution of one km [23]. They permit monitoring of seasonal and inter-annual variation in vegetation status and have been shown elsewhere to be important correlates of the spatial and temporal changes in the distribution of insect vectors of disease [24]. Free access is given to 10 daily synthesized maximum value composite products 3 months after insertion in the VEGETATION archive [25]. Thirty-six decadal NDVI images were obtained from SPOT VEGETATION satellite sensor data archive for 1999. The VEGETATION data used consists of 10 daily NDVI products, compiled from daily synthesis over the previous ten days, for the entire African continent, to which both radiometric corrections and geometric corrections have been applied to enhance product quality. The true value of NDVI was calculated from the 8 bit decadal images (1–255) using ((digital number*0.004)-0.1). From the true values images, mean, minimum, maximum, median, standard deviation were calculated for the year 1999.

In order to take into account the flight distance of the vector, 5 km buffers were created around each study village and the mean value of the NDVI variable was obtained and used in the development of the logistic regression model.

### Validation of the model

Epidemiological data used to validate the model was obtained from a multicentric study supported by TDR which was designed to assess the relationship between clinical and parasitological indicators of *Loa loa *endemicity [15,26]. The surveys were conducted by three research teams (comprising epidemiologists, parasitologists, social scientists and clinicians) based in Buea and Yaoundé, both in Cameroon, and in Calabar, Nigeria. Only data from Cameroon are used in our validation process. The Buea study sites were in South-West and North-West Provinces of Cameroon; in these areas, a total of 4532 individuals over the age of 15 years, living in 42 villages, participated in the survey. The study sites for the Yaoundé team were located in the East Province of Cameroon, where 3181 persons of the same age, living in 32 localities, were examined. A standardized questionnaire was administered to participants from whom finger-prick blood samples were collected and examined for *Loa loa *mfs. Model validation was undertaken through correlating model outputs with the independent data set.

## Results

### Univariate analysis

**Figure 1:**
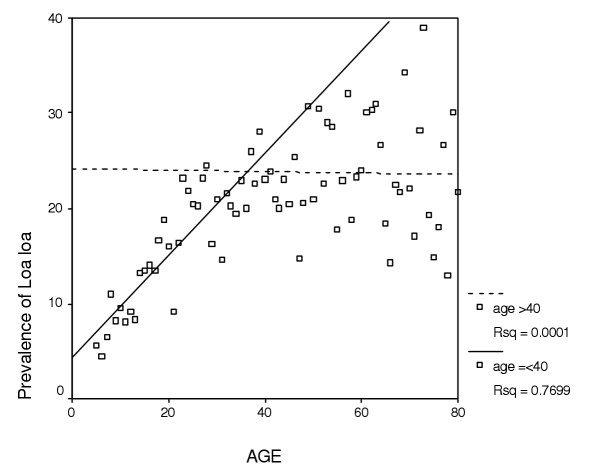
Relationship between prevalence of *Loa loa *microfilaraemia and age.

While *Loa loa *prevalence when regressed against population density was not found to be significantly correlated, a strong and significant linear relationship (*r*^2 ^= 0.7699; *P *< 0.001) was found with age below 40 years (Figure 1). In our analysis we used this linear relationship for all individuals below 40 but then treated all those above 40 as though they remained at this age since any subsequent change with age was deemed non-significant. This broken stick approach was considered to be a reasonable strategy and much simpler than fitting a non-linear model.

Prevalence rates were significantly (*P *< 0.001) higher in males than in females (19.8% n = 6725 and 15.2% n = 7500 respectively). Sex was therefore entered (as a categorical variable) in the logistic regression model.

As one would expect, prevalence rates were significantly higher (*P *< 0.001) in individuals from whom the blood smear was prepared using a volume of 50 μl as compared with those from whom 30 μl of blood were taken. Blood sample size was therefore entered (as a categorical variable) in the logistic regression model.

**Figure 2:**
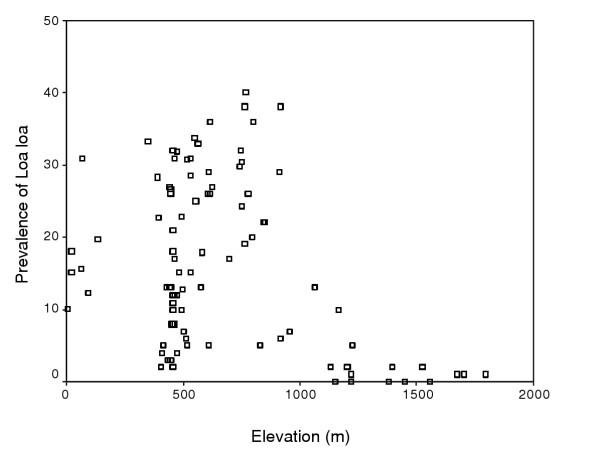
Relationship between prevalence of *Loa loa *microfilaraemia and elevation.

After investigating the value of the DEM and its associated files (slope, aspect, flow accumulation) in predicting microfilaraemia prevalence using univariate and multivariate regression statistics, the DEM alone was chosen as a predictive variable for the development of a logistic regression model. Because the relationship between *Loa loa *prevalence and the DEM was found to be non-linear (Figure 2) the DEM data was divided up into 250 m interval classes and treated as a categorical variable.

**Figure 3:**
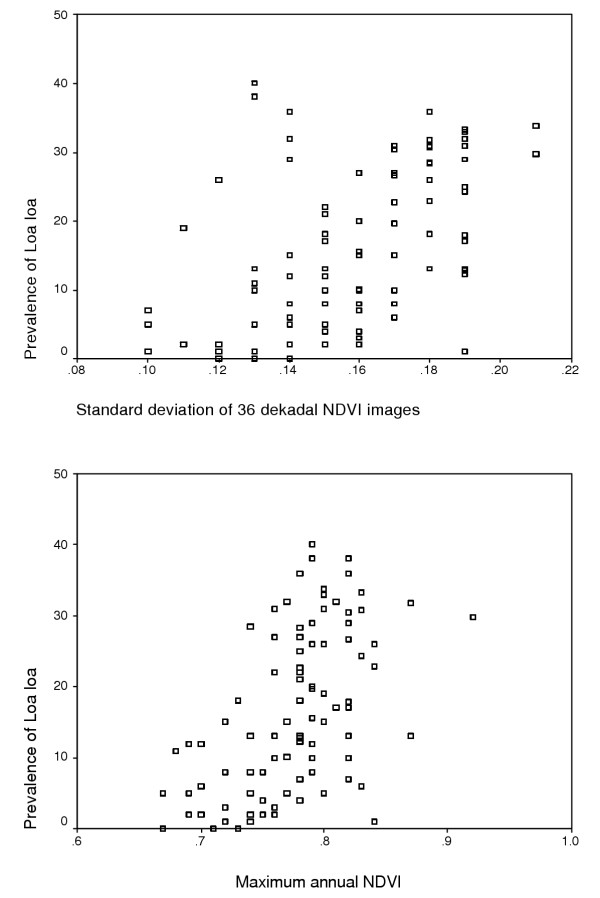
Relationship between prevalence of *Loa loa *microfilaraemia and standard deviation of NDVI (a); and between prevalence of *Loa loa *microfilaraemia and maximum annual NDVI (b).

Since univariate analysis of the SPOT VEGETATION NDVI data against prevalence indicated a non-linear relationship [Figure 3a and 3b], the satellite data were entered into the model as a series of numeric variables (the original, the square and the cube) for the mean, the minimum, the maximum and the standard deviation. Standard deviation and maximum NDVI were most strongly correlated with prevalence using univariate analysis.

### Multivariate analysis

Separate and combined models were created which included all individual level co-variates and altitude for both buffered and non-buffered SPOT VEGETATION data variables. Final model choice was based on (a) the simplicity and biological validity of the model (b) the predictive capacity of the model when assessed using bootstrap methodology (where half of the data was extracted randomly from the data set and tested against model results developed from the remaining half) and (c) the ability of the model to be extended over the large geographic area involved in the APOC programme.

Thus, besides the three individual-level covariates (sex, age and blood sample size), only three village level environmental co-variates were kept in our model: maximum annual NDVI, standard deviation of the NDVI, and elevation. In the final model, the probability of a female individual being infected with *Loa loa *is represented by:

1/(1+e^-*z*^), where (for our example a female aged ≥ 40 years, and a blood sample of 50 μl):

[Z] = ([Sex]*0.221)

+ ([Age] * 0.049)

- ([Blood sample size]*0.542)

+ ([Maximum NDVI^2^] * 74.38)

- ([Maximum NDVI^3^] * 58.816)

+ ([Standard deviation NDVI] * 11.788)

+ (([Elevation] < 250) * 1.133)

+ (([Elevation] = 250-500) * 0.865)

+ (([Elevation] = 500-750) * 0.981)

+ (([Elevation] = 750-1000) * 1.171)

- (([Elevation] = 1000-1250) * 0.623)

- (([Elevation] = 1250-1500) * 1.516)

- (([Elevation] = 1500-1750) * 1.342)

- (([Elevation] = 1750-2000) * 0.669)

- 22.883 (constant),

where [Sex] = 0 for females, and 1 for males; and [Blood sample size] = 0 for 30 μl, and 1 for 50 μl.

Thus for our example, age, sex and blood sample size variables are presented for a fixed value because of their non-spatial nature. The probability of infection increases (+) with increasing value for sex (0 versus 1), with increasing age, and with increasing standard deviation of NDVI and Maximum NDVI^2^; it decreases (-) with Maximum NDVI^3 ^(indicating that the relationship to maximum NDVI is non linear), and increases for categories of elevation between 0 and 1000 metres after which it decreases. The probability of infection decreases with increasing value of blood sample size (0 versus 1) even if larger blood sample should normally allow better detection of the infection. This could be due to the fact that larger samples have been taken often in places where infection rates are low, in the highlands, and this is presumably a result of interacting with the other variables.

**Figure 4:**
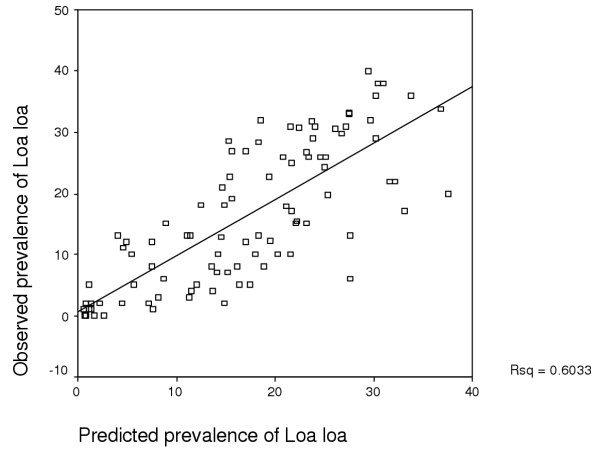
Relationship between observed prevalence of *Loa loa *microfilaraemia and predicted prevalence.

A similar model can be made for males and individuals at any other age. The model was developed using 50% of the data (randomly selected) and then tested on the remaining 50% of the data. When the model results were plotted against the observed data an *r*^2 ^of 0.6033 was obtained (Figure 4). In addition, when one takes the threshold of 20% as the *Loa *microfilaraemia above which there is a risk of post-ivermectin encephalopathy, the sensitivity and specificity of the model (respectively: the proportion of villages with a measured prevalence ≥ 20%, and which were correctly predicted as such by the model; and the proportion of villages with a measured prevalence <20%, and correctly identified as such by the model) were found to be 77.1 and 80.0%, respectively (Table 2).

**Table 2:** Distribution of the villages examined, according to their observed and predicted prevalence of *Loa *microfilaraemia

			**Observed prevalence**	
			<20%	≥ 20%
	
**Predicted prevalence**	First dataset	<20%	48	8
	(collected by IRD-CPC)	≥ 20%	12	27
	Dataset used for validation	<20%	20	5
	(collected by TDR)	≥ 20%	18	31

### Mapping the model results: an Environmental Risk Map for *Loa loa*

In order to standardize the variables relating to individuals (which cannot be mapped) we chose values that best represent the average adult population (above the age of 15) i.e. 50:50 male – female ratio, age 40 and a blood sample of 50 μl. Age 32 was the approximate average age of individuals in the data sets used to develop the model. Using age 40 (the average age of individuals >15 years of age) in the model was therefore likely to slightly over-represent infection in these data sets but the final model would be directly comparable with current policy to exclude individuals below the age of 15 from rapid epidemiological surveys for loiasis [14,15]. In order to create a model, which included both males and females, two separate models were created, one for each sex and the mean of the two model results taken.

### Validation of the model

**Figure 5:**
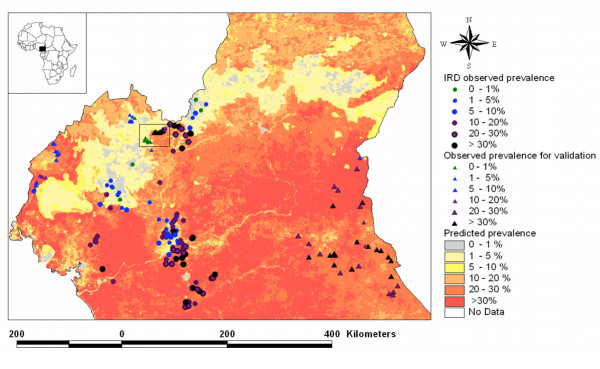
Predictive model of *Loa loa *prevalence for Cameroon overlaid with the observed prevalence data.

**Figure 6:**
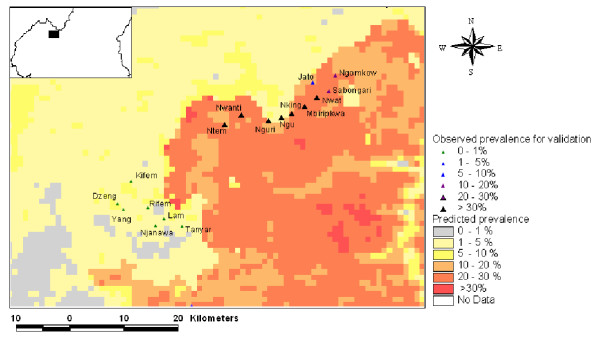
Inset of verification villages in North-West province of Cameroon (note the position of Nguri and Ngu).

Since the chosen model was based on environmental variables, which could be mapped across the entire country, it was possible to extrapolate the model results to the whole of Cameroon (Figure 5) and compare them with the results of the independent TDR survey. Correspondence between observed prevalence from the TDR verification data set and the model results was found to be very close: the sensitivity and the specificity of the model, when using the prevalence threshold of 20%, were 86.1 and 52.6%, respectively. Five villages were observed to have high prevalence rates (>20%) despite being model predictions for prevalence below 20% (Table 2). Two villages, Nguri and Ngu, located in the North-West province, were classified as extremes (Figure 6). Inaccuracies in the georeferencing of the village location or the spatial data used to create the model may account for this, as could the possibility that the local population regularly visit the *Chrysops *infested area nearby. The three other villages (Ntem, Boum and Baktala) are all villages from the Eastern Province of Cameroon which were sited on very localised areas where model predictions for <20% prevalence occurred. These villages were all within 1 km of areas where model results predicted >20% prevalence.

When one considers both datasets as a whole, and the prevalence threshold of 20%, the sensitivity and the specificity of the model were 81.7 and 69.4%, respectively.

## Discussion

Detailed information on distribution of disease and levels of endemicity is a prerequisite for effective planning of control programmes. This has been an important element in the planning of the African Programme for Onchocerciasis Control (APOC) where community-directed treatment with ivermectin (Mectizan^®^) has been targeted at areas of hyper- and meso-endemicity, identified by Rapid Epidemiological Mapping [27]. The expansion of the APOC programme in Cameroon has been delayed due to severe (sometimes fatal) adverse reactions in patients receiving ivermectin who were co-infected with *Loa loa. *This has led to caution in defining new areas for the expansion of the programme and a requirement for identifying areas of high risk of *Loa loa *using a potentially rapid and extensive methodology. Thomson *et al*. [17] developed a preliminary model using satellite mapping based on existing knowledge of distribution and prevalence, and recently a method of rapid assessment using a simple questionnaire, called RAPLOA, has been developed [15].

This paper further develops the satellite-derived risk map of *Loa loa *by using improved satellite data sets and detailed information on infection rates in villages in various ecological zones in Cameroon. The resulting model is able to predict prevalence risk throughout southern Cameroon with a greater accuracy than hitherto available. The final model has been chosen because of the good fit between observed and predicted prevalence and is based on elevation and 1 km SPOT VEGETATION data. In order to understand the value of the risk map it is important to note that (a) while the 1 km resolution of the environmental data cannot reveal the small and localised muddy breeding sites of the *Chrysops *vectors, their general habitat is well captured by the model and it is recognised that the 1 km resolution is much less than the dispersal capacities of the adult flies; and (b) the model is not dependant on the seasonal or inter-annual variability of the vector density, since this is not reflected in fluctuations in *Loa *microfilaraemias, because the adult worm life-span is much longer (4–17 years).

Among the areas in which the prevalences of *Loa loa *are particularly high, according to the Environmental Risk Map (ERM), two should be pointed out, because they are also those where most of the cases of serious adverse events (SAEs) have been recorded so far. The first one is the Lekie Department (Central Province), in the Sanaga valley, where 53 of the 63 probable or possible *Loa loa *encephalopathy cases recorded between 1989 and 2001 in Cameroon have occurred [3]. Besides the high level of endemicity, this cluster of cases may be related to the high population density there, as well as the proximity of the area with the capital, Yaoundé, which probably led to a high reporting rate; however, other hypothesis should be considered, such as specific susceptibility of the local human populations, or special pathogenicity of the local *Loa *"strain" [28,29]. The latter possibility is currently investigated in Cameroon, and in the Mayumbe forest (Democratic Republic of Congo), another area where some 15 cases of fatal SAEs were reported in December 2003. The second area of interest shown by the ERM in Cameroon is the Tikar plain, near Bankim, between the Western, North-West and Adamaoua provinces, where several cases have been recently reported, though the area's vegetation is of shrub savanna type. The ERM also shows those areas where CDTI projects against onchocerciasis may be implemented in a near future, and where the risk of SAEs is probably high: the Eastern and Southern Provinces, and the north-western part of the Littoral Province.

While the use of satellite-derived environmental data for evaluation of disease risk has been developed for several vector borne diseases (malaria, Rift Valley fever, visceral leishmaniasis, tick-borne encephalitis) [24], limited use of such data in public health programmes for the control of infectious diseases has been achieved.

This paper defines a model which identifies areas of potential high risk of severe adverse reactions to ivermectin, and will contribute to APOC's programme development by enabling resources to be effectively targeted to areas deemed at risk. The Technical Consultative Committee of APOC, and the Mectizan Expert Committee have developed a series of recommendations aimed at "facilitating effective detection and management of SAEs following treatment with Mectizan in known and suspected *Loa loa *endemic areas". Three types of mass treatment strategies have been defined, according to the levels of endemicity of onchocerciasis and of loiasis, the latter being defined by the prevalence of *Loa *microfilariae (<20 % versus ≥ 20%) or of history of eye worm passage (<40 % versus ≥ 40%). In those areas where onchocerciasis is meso- or hyperendemic, and the prevalence of *Loa *mfs exceeds 20%, a number of detailed measures should be taken, regarding training of community distributors and medical personnel, availability of medical supplies, duration of distribution, surveillance of the treated populations, and management of the patients who develop a SAE. Though lower, the risk that SAE occur in areas where the prevalence of *Loa *microfilaraemia is lower than 20% is not nil, and thus guidelines have also been developed regarding the strategy to apply in such situations [30].

The model developed is by no means a definitive product but provides a basis for decision making in terms of where rapid epidemiological surveys for loiasis should now be targeted. Modelling in this way permits an iterative process between field epidemiologist and modeller which not only means that decisions are made on the best available data but that such data is updated rapidly as new survey results are entered in the database and the model refined. Models are valuable in so far as they reflect reality. Given the complexity of disease transmission processes where human, parasite, vector and environment interact, it is impossible to think that all relevant factors can be incorporated into a general model which can be applied on a regional scale. What is significant here is the fact that important decisions need to be made now with regard to the likely spatial extent of the distribution of *Loa loa*. As it stands, the model presented here uses environmental features known to be associated with the biology of the vector, is robust for the area of data collection and predicts areas within Cameroon where *Loa loa *has been found in the past. It provides a rapid assessment methodology of areas where adverse reactions to ivermectin may occur and will be further developed in conjunction with the RAPLOA procedure which APOC intends to apply to CDTI areas potentially endemic for *Loa loa *[15]. The next step in the process will be to update the model with new data (e.g. the verification data set) from all the countries where loiasis is endemic, and to explicitly represent uncertainty in the model outputs so that decision makers will be able to assess for themselves the quality of the model results for their area of interest.

## List of abbreviations

APOC African Programme for Onchocerciasis Control

CDTI Community-Directed Treatment with Ivermectin

CPC Centre Pasteur du Cameroun

DEM Digital Elevation Model

ERM Environmental Risk Map for *Loa loa*

GIS Geographic Information System

GPELF Global Programme to Eliminate Lymphatic Filariasis

IRD Institut de Recherche pour le Développement

Mfs Microfilariae

NDVI Normalised Difference Vegetation Index

NOAA-AVHRR National Ocean and Atmospheric Administration – Advanced Very High Resolution Radiometer

RAPLOA Rapid Assessment of the Prevalence and Intensity of Loa infection

SAE Serious Adverse Event

SPOT Satellite Pour l'Observation de la Terre

TDR UNICEF/UNDP/World Bank/WHO Special Programme for Research and Training in Tropical Diseases

UNEP-GRID United Nations Environment Programme-Global Resource Information Database

USGS United States Geological Survey

## Competing interests

None declared.

## Authors' contribution

MCT designed the study, planned the analysis, supervised the modelling, interpreted the results, and wrote the paper.

VO prepared the data, developed the model, prepared the maps, and wrote the paper.

JK and JG collected the field data used for the development of the model.

SW, IT and PE collected the field data used for the validation of the model.

JHR supervised the study thanks to which the validation data were collected.

DHM proposed the study, contributed to its design and interpretation of the results.

MB designed the study, planned the analysis, collected the field data, interpreted the results, and wrote the paper.
